# Genomic Analysis of a Novel Phage Infecting the Turkey Pathogen *Escherichia coli* APEC O78 and Its Endolysin Activity

**DOI:** 10.3390/v13061034

**Published:** 2021-05-31

**Authors:** Sangsang Deng, Qiang Xu, Yajuan Fu, Leiqin Liang, Yan Wu, Fang Peng, Meiying Gao

**Affiliations:** 1 Wuhan Institute of Virology, Chinese Academy of Sciences, Wuhan 430071, China; SangsangDeng@163.com (S.D.); fuyajuann@163.com (Y.F.); liangleiqin@163.com (L.L.); wuyan_81@126.com (Y.W.); 2 University of Chinese Academy of Sciences, Beijing 100039, China; 3 China Center for Type Culture Collection(CCTCC), College of Life Sciences, Wuhan University, Wuhan 430072, China; 2018282040179@whu.edu.cn

**Keywords:** turkey pathogen, *Escherichia coli*, phage genome, endolysin

## Abstract

Due to the increasing spread of multidrug-resistant (MDR) bacteria, phage therapy is considered one of the most promising methods for addressing MDR bacteria. *Escherichia coli* lives symbiotically in the intestines of humans and some animals, and most strains are beneficial in terms of maintaining a healthy digestive tract. However, some *E. coli* strains can cause serious zoonotic diseases, including diarrhea, pneumonia, urinary tract infections, and hemolytic uremic syndrome. In this study, we characterized a newly isolated *Myoviridae* phage, vB_EcoM_APEC. The phage vB_EcoM_APEC was able to infect *E. coli* APEC O78, which is the most common MDR *E. coli* serotype in turkeys. Additionally, the phage’s host range included *Klebsiella pneumoniae* and other *E. coli* strains. The genome of phage vB_EcoM_APEC (GenBank accession number MT664721) was 35,832 bp in length, with 52 putative open reading frames (ORFs) and a GC content of 41.3%. The genome of vB_EcoM_APEC exhibited low similarity (79.1% identity and 4.0% coverage) to the genome of *Acinetobacter* phage vB_AbaM_IME284 (GenBank no. MH853787.1) according to the nucleotide Basic Local Alignment Search Tool (BLASTn). Phylogenetic analysis revealed that vB_EcoM_APEC was a novel phage, and its genome sequence showed low similarity to other available phage genomes. Gene annotation indicated that the protein encoded by *orf11* was an endolysin designated as LysO78, which exhibited 64.7% identity (91.0% coverage) with the putative endolysin of *Acinetobacter baumannii* phage vB_AbaM_B9. The LysO78 protein belongs to glycoside hydrolase family 19, and was described as being a chitinase class I protein. LysO78 is a helical protein with 12 α-helices containing a large domain and a small domain in terms of the predicted three-dimensional structure. The results of site-directed mutagenesis indicated that LysO78 contained the catalytic residues E54 and E64. The purified endolysin exhibited broad-spectrum bacteriolytic activity against Gram-negative strains, including the genera *Klebsiella*, *Salmonella*, *Shigella*, *Burkholderia*, *Yersinia*, and *Pseudomonas*, as well as the species *Chitinimonas arctica*, *E. coli*, *Ralstonia solanacearum*, and *A. baumannii*. An enzymatic assay showed that LysO78 had highly lytic peptidoglycan hydrolases activity (64,620,000 units/mg) against *E. coli* APEC O78, and that LysO78 had lytic activity in the temperature range of 4–85 °C, with an optimal temperature of 28 °C and optimal pH of 8.0, and was active at pH 3.0–12.0. Overall, the results suggested that LysO78 might be a promising therapeutic agent for controlling MDR *E. coli* APEC O78 and nosocomial infections caused by multidrug-resistant bacteria.

## 1. Introduction

*Escherichia coli* is a Gram-negative, nonsporulating, nonmotile bacterium that normally exists in the intestinal flora of humans and animals. However, several strains of *E. coli* are notable clinical and zoonotic pathogens, such as *E. coli* O157:H7 and *E. coli* APEC O78. *E. coli* O157:H7 is a predominant foodborne diarrheal pathogen that can produce Shiga toxins, which can result in certain life-threatening sequelae [1]. *E. coli* APEC is another avian pathogen; it is transmitted by infected poultry and causes colibacillosis [2]. *E. coli* APEC O78 has been isolated from the gelatinous edema in the lungs of turkeys, and annually this strain can cause multimillion-dollar financial losses globally due to its high mortality and consequent decreased production [3].

Currently, antibiotics abuse—not only in the clinical setting but also in poultry feeding—is already a serious problem, as it leads to drug resistance. Exposure to antibiotic-resistant *E. coli* of common zoonotic pathogens in contaminated water or food constitutes a significant threat to public health and safety [4]. Each year, 700,000 people die from infections caused by drug-resistant bacteria, and the number of cases is estimated to reach 10 million per year by 2050 in the absence of new therapeutics [5]. The development of alternative anti-infection modalities has become one of the highest priorities in modern medicine and biotechnology.

Bacteriophages and their lysins are widely believed to be promising antibacterial agents, and they present some unique advantages over antibiotics. Bacteriophages are the most abundant group of biological entities on Earth, with an estimated abundance of 10^31^—10 times greater than that of their host bacteria [6,7]. Therefore, it is relatively easy to isolate new phages that are expected to be environmentally friendly and have no serious side effects. Generally, it can take 10–15 years and over USD 1 billion to develop a new antibiotic and obtain approval from the Food and Drug Administration (FDA) [8]. By contrast, only a few weeks are needed to obtain new phages for newly emerging strains of resistant bacteria. Furthermore, as bacteria to become resistant, relevant phages will also simultaneously evolve with their host. In other words, when a “superbug” appears, there will be a corresponding “superphage” with action against this superbug that may be readily isolated and produced [9].

Endolysins are bacteriophage-derived peptidoglycan (PG)-degrading proteins that allow mature progeny phages to be released from host bacterial cells at the end of phage lytic cycle [10]. In certain situations, the passage of endolysins to access their peptidoglycan target is facilitated by holins, which are small membrane proteins that dissipate the membrane potential or make holes in the membrane to allow entry [11]. Due to the presence of an outer membrane in Gram-negative bacteria, exogenously added endolysins will usually require an outer membrane permeabilizer to increase the permeability of the bacterial outer membrane and gain access to the PG [12]. Endolysins of Gram-positive bacterial phages usually contain two functional domains—an N-terminal catalytic domain and a C-terminal cell-wall-binding domain [13]. The catalytic domains are peptidoglycan hydrolases, which include the families of N-acetylglucosaminidases, N-acetylmuramidases (lysozymes), N-acetylmuramoyl-L-alanine amidases, and endopeptidases [14]. Endolysin has recently captured the interest of researchers due to its potential as an antimicrobial agent in many areas; thus, the characteristics and applications of endolysins have been the subject of numerous studies. Park et al. reported that endolysin LysECP26, derived from rV5-like phages, could lyse *E. coli* O157:H7 using a lysozyme-like catalytic domain, and that endolysin exhibited strong activity with a broad lytic spectrum against various Gram-negative strains [15]. Park et al. reported that *Salmonella* phage SPN1S endolysin had unusual structural and functional features compared with other endolysins from phages that infected Gram-negative bacteria [16]. Shavrina combined *E. coli* phage T5 endolysin (L-alanyl-D-glutamate peptidase) and polymyxin B (0.4 µg/mL) or chlorhexidine (0.5 µg/mL) to reduce the number of *E. coli* CFUs by five orders of magnitude [17]. Endolysin LysAB54 showed bactericidal activity against multidrug-resistant *A. baumannii* and *P. aeruginosa*, *K. pneumoniae*, and *E. coli* [18]. The phage D2 endolysin Abtn−4 contains an amphipathic helix, and was found to have broad activity against Gram-negative strains [19]. Endolysins have applications in food safety and environmental decontamination, and as effective antimicrobials against drug-resistant bacteria [12,20,21,22].

*E. coli* is responsible for a wide range of diseases, both intestinal (diarrhea) and extraintestinal (urinary tract infection (UTI), septicemia, pneumoniae, and meningitis), making it an ideal target for phage therapy [23]. In the future, coliphages will be valuable alternative pharmaceutical preparations to antibiotic treatments against urinary tract infections (UTIs), which are caused by uropathogenic strains, particularly MDR and extensively drug-resistant (XDR) *E. coli* clinical isolates [24]. Even though more than 314 *E. coli* phage genomes have been uploaded to the National Center for Biotechnology Information (NCBI), only one turkey pathogen *E. coli* APEC O78 phage (vB_EcoM_ECOO78) genome has been studied. Maram et al. evaluated the efficacy of bacteriophage treatment in reducing *E. coli* APEC O78 replication in the avian respiratory tract in vivo. However, the genome information or any other characters of this phage have not been reported [25]. Thus, it is of great significance to discover and study *E. coli* bacteriophages. In this study, we characterized the genome of a novel phage vB_EcoM_APEC and found a novel type of endolysin, LysO78, which was capable of peptidoglycan hydrolases activity against Gram-negative strains. Its broad-spectrum and high bacteriolytic activity over a wide range of pHs and temperatures suggest that LysO78 might be a useful antimicrobial agent.

## 2. Materials and Methods

### 2.1. Bacterial Strains and Culture Conditions

The drug-resistant strains *E. coli* APEC O78 and CC11 were kindly donated by Liancheng Lei’s laboratory (College of Veterinary Medicine, Jilin University, Jilin, China). The other strains used in this study were provided by the China Center for Type Culture Collection (CCTCC, College of Life Sciences, Wuhan University, Wuhan, China), the BeNa Culture Collection (BNCC), and our laboratory. The preservation of the strains was achieved using 20% glycerol stocks at −80 °C and vacuum freeze-drying technology. *E. coli* strains were subjected to shake culturing at 37 °C in Luria–Bertani (LB medium). *Chitinimonas arctica* R3-44^T^ was grown at 28 °C in R2A. *E. coli* BL21 (pET28a) was used to express LysO78.

### 2.2. Phage Isolation and Morphology Observation

The *Chitinimonas arctica* R3-44^T^ colonies were inoculated in R2A broth and shaken to the exponential phase (OD600 = 0.6) at 28 °C, then mitomycin C (Sigma) was added for prophage induction at a final concentration of 0.5 μg/mL. Following induction, the supernatant was collected by centrifugation and filtration sterilization using a 0.22 µm pore size membrane filter after culturing for 8 h. *C. arctica* R3-44^T^, *C. viridis* KCTC 22839, *E. coli* CC11, and *E. coli* APEC O78 were used as indicator strains to isolate phages. A standard double-layer plaque assay was used to identify plaques. *E. coli* APEC O78 was used for phage propagation. The phages were purified using a CsCl density gradient (1.32, 1.45, 1.50, and 1.70 g/mL) with centrifugation at 25,000× *g* for 4 h at 4 °C. CsCl was removed from the purified phages by dialysis. The induced supernatant of R3-44^T^ and purified phage morphologies were investigated by absorbing phages onto copper–formvar–carbon grids for 10 min, followed by negative staining for 1 min with 2% phosphotungstic acid (PTA, pH 7.0). The grids with adsorbed phages were dried in air and observed under transmission electron microscopes (TEM) at an acceleration voltage of 100 kV (TEM, H-7000FA, HITACHI, Tokyo, Japan).

### 2.3. Host Range Investigation and EOP Analysis of Phage

To investigate the host range of phage vB_EcoM_APEC, a panel of strains belonging to the genera *Salmonella, Shigella, Klebsiella, Pseudomonas, Escherichia, Acinetobacter, Yersinia, Ralstonia, Burkholderia, Bacillus, Listeria, Mycobacterium, Chitinimonas, Streptococcus,* and *Staphylococcus* were attested for phage infection. Each strain was grown in optimal culture conditions (including the medium) according to the specific instructions of the strain-preservation institutions. The host range determination of phage vB_EcoM_APEC was performed with the double-layer overlay assay method according to the procedure described by Mirzaei and Nilsson [26]. To generate phage plaques, 200 µL of the exponential growth cultures of each strain and 100 µL of each diluted phage stock (the five phage lysates were diluted 10^4^–10^8^ times from the phage stock of a titer of 1 × 10^8^ plaque-forming units (PFU)/mL), mixed gently, and then incubated at 37 °C for 60 min). Then, the prepared mixture was added into 5 mL melted semisolid medium (0.3% agarose) with a temperature of 45 °C and overlaid onto a solid medium agar plate. Then, the plate was cultivated for a period of up to 48 h to observe the formation of phage plaques. All experiments were carried out in triplicate.

To detect the efficiency of plating (EOP) of the phage vB_EcoM_APEC on the susceptible strains, the bacteriophage plaques were counted and the average PFU calculated. The EOP of the optimal host, *E. coli* APEC O78, was taken as 100.0%. The target strain EOP = (average PFU on target strain/average PFU on the optimal host strain) × 100%. All experiments were carried out in triplicate.

### 2.4. Phage Genome Sequence and Analysis

The genomic DNA of the phage vB_EcoM_APEC was extracted via phenol–chloroform extraction with proteinase K–sodium dodecyl sulfate (SDS) treatment [27]. The purified phage genomic DNA was sequenced using PromethION from the Oxford Nanopore Technologies (ONT) sequencing platforms. The software Flye 2.6 was used to assemble clean high-quality reads into contigs, and gaps between contigs were filled by primer walking to obtain complete genome sequences [28]. The coding sequences (CDSs) in the genome were predicted using the RAST and FGENE SV software by visual verification [29,30]. Each predicted gene was annotated by performing a search in the NCBI nonredundant protein sequences (NR) and conserved domain (CDD) databases using the Basic Local Alignment Search Tool (BLAST) [31], combined with an analysis of the motif and functional domain composition of the predicted protein using the Pfam 32.0 database [32], HHpred database [33], SMART [34], and EMBL-EBI search [35]. Transfer and ribosomal RNA (tRNA and rRNA) genes were identified using tRNAscan-SE-1.23 and RNAmmer 1.2 server, respectively [36,37]. A phylogenetic analysis of the proteins was performed using MEGA X with the neighbor-joining method and a bootstrap analysis (1000 replicates) with ClustalW alignment [38].

### 2.5. Bioinformatic Analysis of the Putative Endolysin Gene from Phage vB_EcoM_APEC

The nonredundant protein database was searched using the protein BLAST (BLASTP) with the amino acid sequences of endolysin LysO78 as the query [31]. Protein Similarity Search (PSI-Search) was also used to find distantly related protein sequences of endolysin LysO78 [35]. The amino acid sequences of LysO78 and several known endolysins were aligned using ClustalW2 [39] and manually adjusted. WebLogo 3 was used to generate a graphical representation of the amino acid multiple-sequence alignment result [40,41]. Functional domains were searched against the Pfam database [32] and CDD [42]. The three-dimensional structure was predicted using the server Phyre2 [43]. Structural analyses were carried out using Coot [44]. The protein structural figures presented in this study were generated using PyMOL [45].

### 2.6. Expression and Purification of Endolysin LysO78

The *lysO78* gene (open reading frame 11 (ORF11)) was amplified from the phage vB_EcoM_APEC genome using the primer pairs endolysin-For/*Bam*HI (5′-CGCGGATCCGCGATGATCATGACAGAGAAAGGC-3′) and endolysin-Rev/*Hind*III (5′-CCCAAGCTTGGGTTA TGGCTGGCGCAAAGCCTT-3′) and inserted into the vector pET28a to construct the recombinant plasmid pET28a/lysO78. The recombinant plasmid was transformed into *E. coli* BL21 (DE3), and the positive transformants were confirmed by Sanger DNA sequencing, and then selected for their expression of LysO78. The *E. coli* BL21 (DE3) harboring the endolysin vector was grown in 3 L LB supplemented with 50 mg/mL kanamycin to an optical density (OD_600nm_) of 0.6 (4 h, 200 rpm at 37 °C). Recombinant protein expression was induced by isopropyl-β-D-thiogalactopyranoside (IPTG) to a final concentration of 0.4 mM for 5 h at 20 °C. The culture was then centrifuged at 8000 rpm for 10 min, and the cells were disrupted by resuspending the pellet in 120 mL lysis buffer (50 mM Tris-HCl, 300 mM NaCl, 10% glycerol, pH 8.0). The cells were then lysed using a nano homogenizer machine (ATS Engineering Inc., AH1500) for 4 cycles (600 bar). Insoluble cell debris was removed by centrifugation (10,000 rpm, 60 min, 4 °C). Then, the supernatant was filtered (0.22 µm filters) and loaded in a 5 mL HisTrap HP column (GE Healthcare, Waukesha, WI, USA) stacked with Ni/nitrilotriacetic acid (Ni^2+^-NTA) resin for purification. The washing buffer (50 mM Tris-HCl, 300 mM NaCl, 40 mM imidazole, 10% glycerol, pH 8.0) was used for removing nonspecific proteins, and elution buffer (50 mM Tris-HCl, 300 mM NaCl, 160 mM imidazole, 10% glycerol, pH 8.0) was used to elute the target protein. The eluted target protein was then further purified using a G75 sephadex column with lysis buffer, and the protein concentration was determined using the BCA Protein Assay Kit with bovine serum albumin (BSA) as a standard (Thermo Scientific, Waltham, MA, USA). Pure protein was stored at 4 °C for further use. A protein molecular weight calculator was used to analyze the molecular weight of recombinant LysO78 [46].

### 2.7. Functional Analysis of Catalytic Residues

To validate the functional assignment of E54 and E63 (or E64) as a catalytic dyad in endolysin LysO78, we performed an antimicrobial activity assay using various mutants of LysO78, in which outer-membrane-permeabilized *E. coli* APEC O78 was used as a substrate. Wild-type and mutant LysO78 were added into the bacterial suspension, with incubation for 30 min. The changes in the absorbance of the strain at 600 nm were determined. The homologous recombinant primers of single amino acid site mutants (E54A, E54Q, E54D, E63A, E63Q, E64A, E64Q) and double site mutants (E54A/E63A, E54Q/E63Q, E54A/E64A, E54Q/E64Q) were designed in CE Design (Vazyme Biotech Co., Ltd., Najing, Jiangsu, China) for PCR amplification (Table S2). The mutants were constructed using the Mut Express^®^ II Fast Mutagenesis Kit V2 (Vazyme Biotech Co., Ltd. Najing, Jiangsu, China; article number: C214-01). The positive transformants were identified by Sanger DNA sequencing, and the right mutants were expressed and purified using the same method described in Section 2.6. All experiments were carried out in triplicate.

### 2.8. Lytic Activity Assay of LysO78

To analyze the optimum concentration of the protein LysO78, *E. coli* APEC O78 in the exponential growth phase was collected by centrifugation at 8000 rpm for 3 min at 10 °C, and the pellets were washed twice using phosphate-buffered saline (PBS, pH7.4) and then resuspended in PBS buffer to adjust the turbidity to 0.8 (optical density at 600 nm (OD_600_)). The endolysin LysO78 with different concentration gradient was added into the bacterial suspension. Ethylenediaminetetraacetic acid (EDTA) was added into the above reaction solution to a final concentration of 50 mM to increase the permeability of the bacterial outer membrane [47]. We measured and calculated the endolysin LysO78 activities according to the previously described method [48]. The samples without enzyme were set as negative controls. For the purpose of testing the optimal lytic pH, we modified a previously described method [49]. Briefly, *E. coli* APEC O78 in logarithmic growth phase was washed and then resuspended in buffers of different pH, including pH 3.0, 4.0, 5.0, and 6.0 (50 mM Na_2_HPO_4_–citric acid, 300 mM NaCl, and 10% glycerol); pH 7.0, 8.0, 9.0, and 10.0 (50 mM Tris–HCl, 300 mM NaCl, and 10% glycerol); and pH 11.0 and 13.0 (50 mM Na_2_CO_3_–NaOH, 300 mM NaCl, and 10% glycerol). To determine the optimal reaction temperature of LysO78, the strain *E. coli* APEC O78 was washed and resuspended in PBS, then LysO78 was added into the suspension to incubate the respective mixtures at 4, 16, 28, 37, 45, 55, 65, 75, and 85 °C for 30 min. The changes in the absorbance of the strain at 600 nm were determined. To analyze the endolysin’s thermostability, LysO78 in buffer (50 mM Tris–HCl, 300 mM NaCl, and 10% glycerol, pH = 8.0) was first treated at different temperatures (4, 16, 28, 37, 45, 55, 65, 75, and 85 °C) for 1 h, and the lytic activity was then assayed using the method described above. All experiments were carried out in triplicate.

### 2.9. The Host Range Activity Spectrum of Endolysin LysO78

To investigate the host range activity spectrum of endolysin LysO78, the lytic activities against the genera *Salmonella*, *Shigella*, *Klebsiella*, *Pseudomonas*, *Escherichia*, *Acinetobacter*, *Yersinia*, *Ralstonia*, *Burkholderia*, *Bacillus*, *Listeria*, *Mycobacterium*, *Chitinimonas*, *Streptococcus*, and *Staphylococcus* were assayed. For testing the lytic activity against Gram-negative strains, EDTA was added to the reaction mixture to a final concentration of 50 mM to increase the permeability of the bacterial outer membrane [50]. In addition, the lytic activity of the LysO78 on *C. arctica* R3-44^T^ was also assayed by changing the experimental conditions, as previously described [51]. Chloroform-saturated Tris buffer (0.05 M, pH 7.7) was used to remove the outer membrane of the R3-44^T^ cell wall and exposed the peptidoglycan layer to LysO78 adequately. The changes in bacterial concentration were determined by detecting the turbidity of the bacterial suspension at 600 nm. All experiments were carried out in triplicate.

### 2.10. PCR Screening for the Occurrence of vB_EcoM_APEC Encoding Genes

An inoculation loop was used to collect *C. arctica* R3-44^T^ colonies from a lawn grown on a solid R2A plate and transferred into 100 μL of ddH_2_O, then the mixture was boiled for 15 min and immediately placed on ice for 5 min. The bacterial suspension was then centrifuged at 13,000 rpm for 5 min at 4 °C, and the supernatant was used as a DNA template for PCR. The occurrence of vB_EcoM_APEC encoding genes was evaluated by PCR for major capsid genes (*orf50*, 891 bp) using the primer pairs Capsid-For (5′-ATGACAGCAGATACTATTAA-3′) and Capsid-Rev (5′–TTAAGCACTCAAGAACTCAA-3′). Positive and negative controls using the phage genome and ddH_2_O as templates, respectively, were included for all PCRs. Meanwhile, the detection of the 16S rDNA gene was carried out to evaluate the DNA availability of different colonies using the primers 27-For (5′ AGAGTTTGATCCTGGCTCAG 3′) and 1492-Rev (5′ TACGGTTACCTTGTTACGACTT 3′).

## 3. Results

### 3.1. Isolation and Morphology of Phage vB_EcoM_APEC

**Figure 1:**
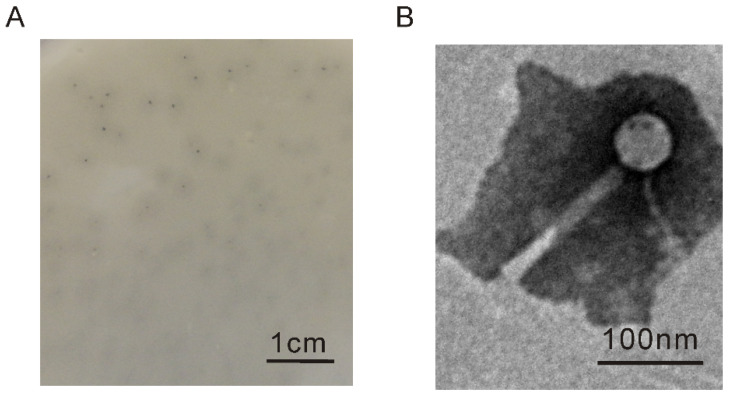
The morphology characteristics of phage vB_EcoM_APEC. (**A**) Plaque morphology of phage vB_EcoM_APEC; (**B**) virion morphology observation of phage vB_EcoM_APEC under TEM.

The prophages of *C. arctica* R3-44^T^ were induced using mitomycin C, and *Myoviridae* and *Siphoviridae* phages were observed in the induced supernatant using TEM (Figure S1). The supernatant of induced strain R3-44^T^ could contain phage plaques formed by using the turkey pathogenic *E. coli* APEC O78 as an indicator strain, but no phage plaques were observed on the double-layer plates of other indicator bacteria, including *C. arctica* R3-44^T^, *C. viridis* KCTC 22839, and *E. coli* CC11. A phage designated as vB_EcoM_APEC was isolated from the plaque of *E. coli* APEC O78 double-layer plates. The phage vB_EcoM_APEC formed tiny plaques surrounded by semitransparent halos (Figure 1A). This halo-like appearance might suggest the production of a native depolymerase from the phage [52]. Based on morphological identification via TEM, vB_EcoM_APEC was revealed to be a member of the *Myoviridae* family with an icosahedral head (height, 53.1 nm ± 1.2 nm; width, 52.5 nm ± 1.1 nm) and a contractile tail (length, 146.3 nm ± 1.4 nm; width, 14.8 ± 0.5 nm) (Figure 1B). The morphologic comparison showed that one of the induced phages from the supernatant of *C. arctica* R3-44^T^ was similar to the phage vB_EcoM_APEC (Figure S2).

### 3.2. Host Range of Phage vB_EcoM_APEC

The host range of phage vB_EcoM_APEC was investigated by infecting 60 bacterial strains of different genera and species. The results shown in Table 1 indicate that phage vB_EcoM_APEC was able to lyse not only the avian pathogenic strain *E. coli* APEC O78, but also *E. coli* K12 and *K. pneumoniae* subsp. *ozaenae*, which are considered the most common causative agents, with a high virulence and the cause of the antibiotic resistance of hospital-acquired pneumonia in the United States. *E. coli* APEC O78 was the optimal host, and we assumed that its EOP was 100.0%; the EOP of *E. coli* K12 and *K. pneumoniae* subsp. *ozaenae* was 76.7% and 51.9%, respectively.

### 3.3. General Genomic Characteristics of Phage vB_EcoM_APEC

**Figure 2:**
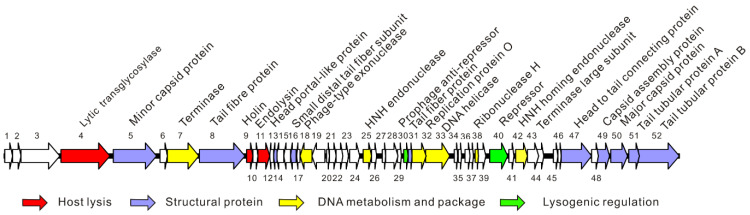
Schematic diagram of the genome of phage vB_EcoM_APEC. The different colors indicate the functional classification of the genes.

The complete genome of phage vB_EcoM_APEC (GenBank no. MT664721) was 35,832 bp, and the GC content was 41.3%. The genome of phage vB_EcoM_APEC encodes 52 ORFs, of which 25 ORFs (48.1%) were matched with known functional proteins, including structural proteins, lysogenic regulatory proteins, host lysis proteins, and DNA replicative and package proteins (Figure 2). No rRNA and tRNA genes were annotated in the genome. Pfam and CDD analyses indicated that ORF4 (putative exolysin) was a mannosyl-glycoprotein endo-β-N-acetylglucosamidase belonging to glycoside hydrolase family 73. The domain architectures of protein ORF4 analyzed by SMART showed that the protein contained one glucosaminidase domain (345–495 amino acids (aa), 777–862 aa) and one bacterial periplasmic substrate-binding domain (PBPb, 128–342 aa). The host lysis system consisted of the predicted proteins ORF9 (holin) and ORF11 (endolysin). The function of ORF11 (putative endolysin) is to lyse the host cell and release phage progeny. The holin protein cooperates with endolysin by forming pores on the host bacterium’s cell membrane to deliver the endolysin to the cell wall of the host. ORF5, ORF8, ORF13, ORF16, ORF31, ORF47, ORF49, ORF50, ORF51, and ORF52 were identified as structural proteins of phage vB_EcoM_APEC (Table S1). Both ORF30 and ORF40 contained a classical Cro/CI-type helix-turn-helix (HTH) DNA-binding domain, and were predicted to be a phage antirepressor and repressor, respectively. Hence, they probably control the switching from a lysogenic to lytic cycle [53].

### 3.4. Genomic Comparison and Phylogenetic Analysis of Phage vB_EcoM_APEC

**Figure 3:**
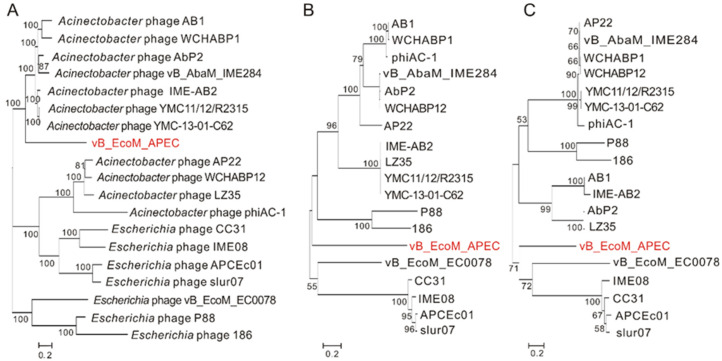
Phylogenetic relationship of phage vB_EcoM_APEC with other phages that infect members of the genera *Acinetobacter* and *Escherichia*. Neighbor-joining trees were constructed on the basis of complete genome sequences and amino acid sequences using MEGA X with 1000 bootstrap replicates. (**A**) Phylogenetic tree based on the whole-genome sequences; (**B**) phylogenetic tree based on the amino acid sequences of major capsid proteins; (**C**) phylogenetic tree based on the amino acid sequences of the terminase large subunits.

Nucleotide BLAST (BLASTn) showed that the genome of vB_EcoM_APEC exhibited low similarity (79.1% identity and 4.0% coverage) to the genome of *Acinetobacter* phage vB_AbaM_IME284 (GenBank no. MH853787.1) and had no significant similarity to the genome of *E. coli* APEC O78 phage vB_EcoM_ECOO78. The major capsid protein of vB_EcoM_APEC exhibited low identity (38.5% identity and 97.0% coverage) to the major capsid protein of *Caudovirales* phage (GenBank no. AXH71877.1). Phage vB_EcoM_APEC revealed low identity to *Acinetobacter* phage YMC-13-01-C62 LD30_gp14 (48.9% identity and 74.0% coverage, YP_009055435.1) and *Pectobacterium* phage MA13 (50.0% identity and 36.0% coverage, QGF20964.1) on the terminase large subunit. According to the phylogenetic analysis of the complete genome sequences and amino acid sequences of the conserved proteins (major capsid protein and terminase large subunit), phage vB_EcoM_APEC belongs to a new phage lineage (Figure 3), indicating that vB_EcoM_APEC is a novel genus of the *Myoviridae* family.

### 3.5. Bioinformatic Analysis of Endolysin LysO78

**Figure 4:**
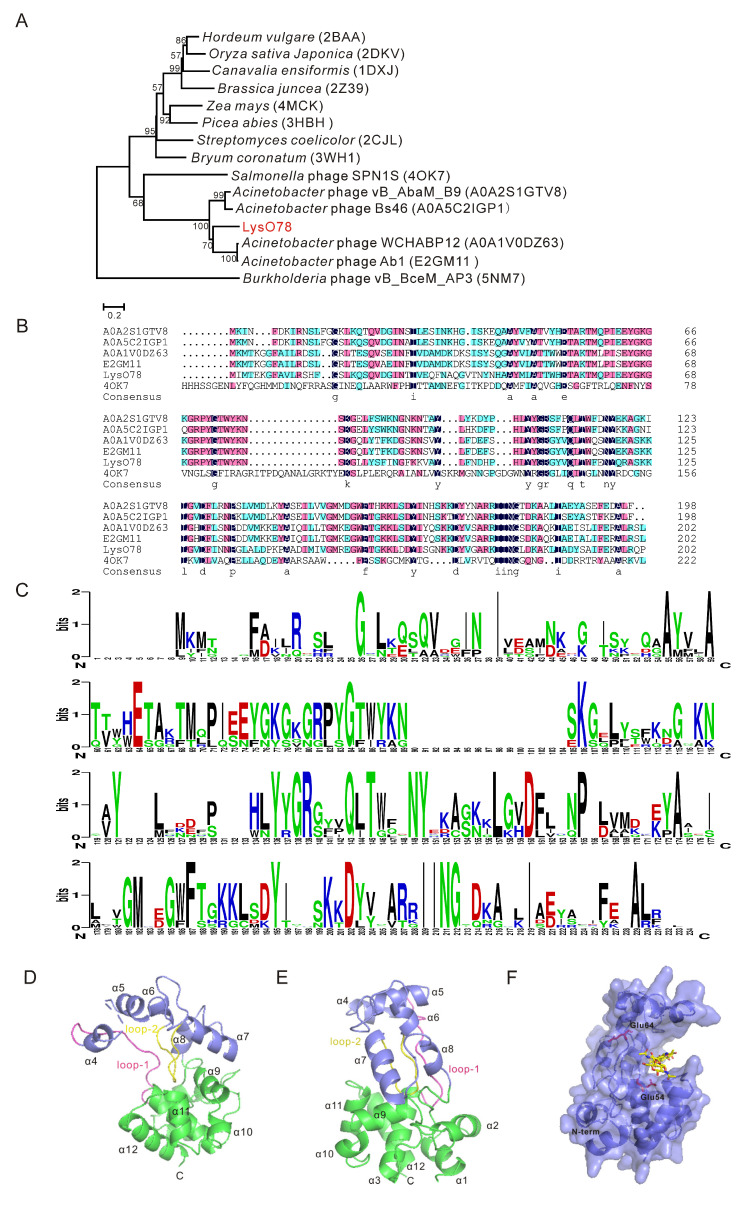
Bioinformatic analysis of the endolysin LysO78. (**A**) Phylogenetic analysis of LysO78 with other phage endolysins and plant chitinases. The proteins used for the phylogenetic analysis were chosen through sequence and structure similarity. The most similar proteins in terms of sequence are the *Acinetobacter* phages endolysins, shown by their UniPortKB identifier (ID). (**B**) Amino-acid sequences alignment of LysO78 with *Acinetobacter* bacteriophage endolysins and *Salmonella typhimurium* phage SPN1S endolysin (PDB ID: 4OK7). The amino acid homology equal to 100% are colored in deep blue; the amino acid homology equal or overtop to 75% are colored in magenta; the amino acid homology equal or overtop to 50% are colored in cyan. (**C**) Sequence conservation analysis of LysO78 and other phage endolysins. The basic amino acids (K, R, H) are colored in blue; the acidic amino acids (D, E) are colored in red; the nonpolar hydrophobic amino acids (A, V, L, I, P, W, F, M) are colored in black; the polar uncharged amino acids (C, G, Q, N, S, Y, T) are colored in green. (**D**,**E**) The overall structure of endolysin LysO78 is shown in two different orientations. The left panel presents a side view, while the front view is shown in the right panel. The large domain and small domain are colored in green and slate, while the two groove loops are shown in magenta and yellow. (**F**) The structure of endolysin LysO78 complexed with a pseudosubstrate. The overall structure is shown in cartoon and surface representations colored in blue and slate. The catalytic residues, E54 and E64, are shown in stick representation and colored in magenta, and the (GlcNAc)_4_ molecule is shown in stick representation and colored in yellow.

Using position-specific iterated BLAST (PSI-BLAST) analysis, *orf11* was identified as an endolysin-coding gene, and the encoded protein was named LysO78. Though the multiple-sequence alignment results showed that LysO78 had relatively high identity with the endolysins of *Acinetobacter* phage vB_AbaM_B9 (64.7% identity with 91.0% coverage), *Acinetobacter* phage WCHABP12 (62.0% identity and 99.0% coverage), *Acinetobacter* phage AB1 (61.5% identity and 99.0% coverage), and *Acinetobacter* phage BS46 (63.6% identity and 91.0% coverage) (Figure 4B), the functions of these similar *Acinetobacter* phage endolysins have not been reported in previous studies. LysO78 exhibited low identity (32.5% identity and 43.0% coverage) with the phage SPN1S endolysin (Protein Data Bank (PDB) identifier (ID): 4OK7) [16] (Figure 4A). The Pfam and CDD analysis results showed that LysO78 belongs to the chitinase class I type group, and that it is a putative member of glycoside hydrolase (GH) family 19 and the broad lysozyme-like superfamily according to the endolysin homology classification. The sequence conservation analysis of LysO78 revealed that the highly conserved residues E54, E63, E64, Q112, L113, T114, N118, I179, N180, and D183 might be important for catalytic activity or substrate binding (Figure 4C).

LysO78 showed structural similarity to the proteins of glycoside hydrolase (GH) family 19. *S. typhimurium* phage SPN1S endolysin, *Picea abies* class IV chitinase (PDB ID: 3HBH) [54], *Streptomyces coelicolor* chitinase (PDB ID: 2CJL) [55], and *Bryum coronatum* chitinase (PDB ID: 3WH1) [56] were used for further comparison. The structural similarity of endolysin LysO78 to 4OK7 (root-mean-square deviation (RMSD)~0.7 Å, 23.0% identity), 3HBH (RMSD~2.1 Å, 23.0% identity), 2CJL (RMSD~2.4 Å, 24.0% identity), and 3WH1 (RMSD~2.3 Å, 23.0% identity) was analyzed using the Dali server algorithm [57]. LysO78 had the same structural components as 4OK7, 3HBH, 2CJL, and 3WH1. According to the structural prediction result for Phyre2, LysO78 had the closest structure to the phage SPN1S endolysin. The overall structure of LysO78 (I2–R199) contained 12 α-helices (Figure 4D,E). These helices formed two domains, including a large domain (I2–T55 and P139–R199; α1–α3 and α9–α12) and a small domain (A56–D138; α4–α8). A substrate-binding groove found between the two domains was composed of two loops—an interdomain connecting loop between α3 and α4 (designated “loop-1”) and another U-shaped loop in the small domain between α6 and α7 (designated “loop-2”) (Figure 4D). According to its structural superimposition with the SPN1S endolysin, the endolysin LysO78 contained putative catalytic residues E54 and E63, while the sequence conservation analysis of LysO78 and *Acinetobacter* phage endolysins revealed that E64 was more highly conserved than E63. Substrate (GlcNAc)_4_ binding to the deep groove was simulated through the structure of *Bryum coronatum* (BcChi-A) chitinase in complex with the substrate (PDB ID: 3WH1) (Figure 4F).

### 3.6. Functional Analysis of Catalytic Residues of Endolysin LysO78

**Figure 5:**
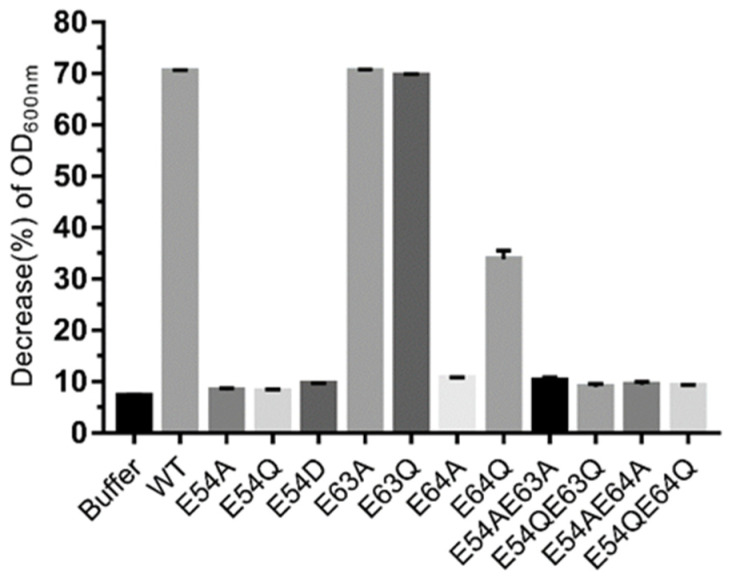
Lytic activity of endolysin LysO78 and its mutants. The antimicrobial activity assay was performed using the WT endolysin LysO78 and various mutant enzymes. Absorbance at 600 nm was measured using *E. coli* APEC O78 as a substrate. Measurements were performed in triplicate for each sample and error bars were calculated from these measurements. The decrease (%) in optical density at 600 nm (OD600) = (1 − absorbance of the bacterial suspension at the end of each treatment/absorbance at the beginning of each treatment) × 100%.

Based on the results of the bioinformatic analysis above, the residues E54, E63, and E64 were selected for site-directed mutagenesis to further confirm the catalytic activity of these sites. The results showed that mutants of endolysins LysO78, including single amino acid site mutants (E54A, E54Q, E54D, E64A) and double site mutants (E54A/E63A, E54Q/E63Q, E54A/E64A, E54Q/E64Q) were essentially inactive (Figure 5). However, the mutant E64Q could maintain almost 50.0% of the lytic activity of wild endolysin LysO78. This might be due to the fact that glutamine (Q) has a similar chemical structure to glutamic acid (E) and represents a more conservative mutation—namely, that Q could retain some lytic activity to a certain degree. Besides this, all other mutant endolysins such as E63A and E63Q maintained a lytic activity equivalent to the wild type of LysO78, indicating that residue E63 identified in the loop was not the catalytic residue of the enzyme. Therefore, according to the bioinformatic analysis and site-directed mutagenesis data, E54 and E64 most likely served as catalytic residues for endolysin LysO78.

The bioinformatic analysis results indicated that E54 was highly conserved and E64 was relatively conserved. The catalytic mechanism of LysO78 may be analogous to the single displacement mechanism of the family 19 chitinases. The catalytic reaction in the family 19 chitinases requires two acidic residues, one acting as a general acid to donate a proton to the β-(1, 4) glycosidic oxygen linking two adjacent glycosides, and the other as a general base, activating water for a concerted nucleophilic attack at C1′ [58]. E64 serves to charge stabilize the oxocarbenium ion in addition to recruiting and activating a nucleophilic water. A plausible explanation for the inactivity mutants of E54A and E54Q might be the side-chain polarity change while E54D was disadvantaged in a three-dimensional structure, owing to the reduction of a methyl group (–CH_2_). A possible reason for the inactivity of the mutant E64A might be its lack of a side chain to charge-stabilize the oxocarbenium ion, while E64Q had a carboxylic acid group (–COO–), which helped it to retain 50.0% lysis activity.

### 3.7. Lytic Activity of the Endolysin LysO78

**Figure 6:**
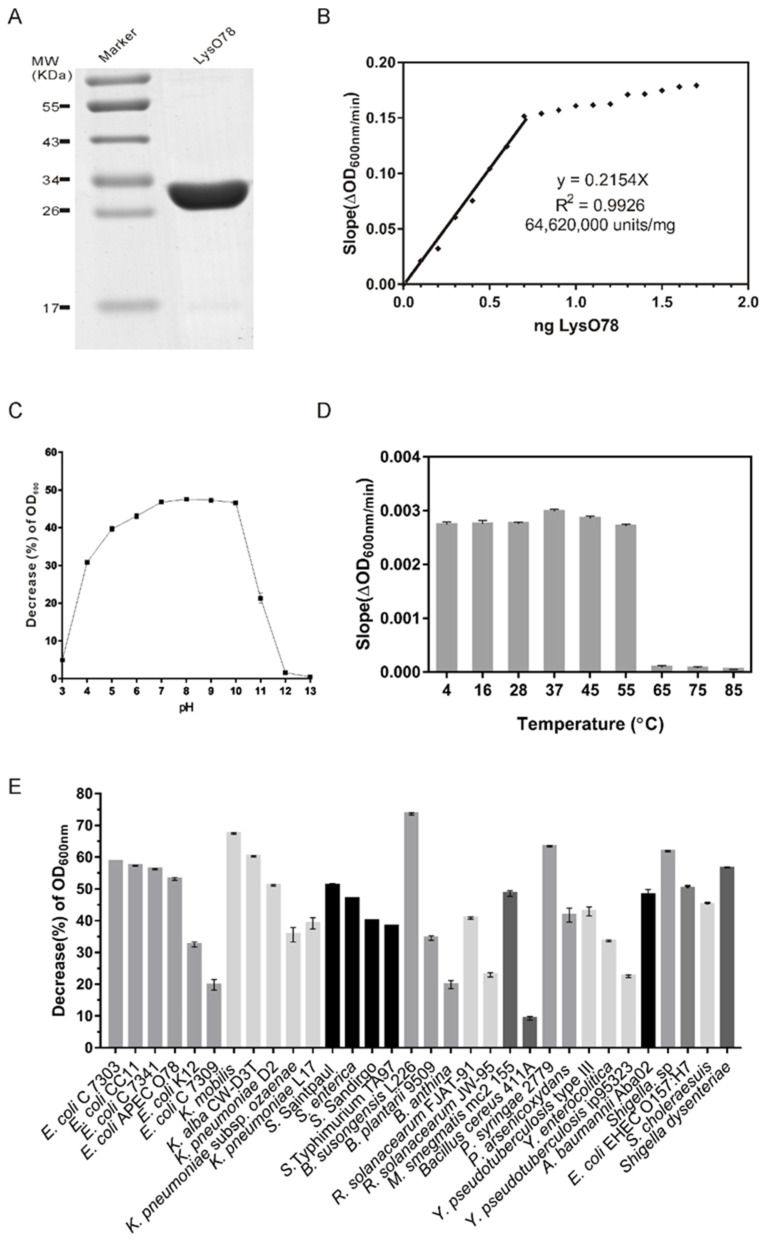
The purification and lytic activity of the endolysin LysO78. (**A**) Purified LysO78. (**B**) Saturation curve of the endolysin LysO78 activity. The X and Y axes display the amount of endolysin added and the corresponding activity measured, respectively. Each data point corresponds to the average value of triplicate samples. The linear region was calculated by the maximalization of the determination coefficient (R²) of the linear regression, and the corresponding slope was a measure of the total endolysin LysO78 activity (units/mg). (**C**) The influence of pH on the lytic activity of LysO78. (**D**) Temperature stability of endolysin LysO78. Proteins were initially treated at different temperatures for 1 h and then the lysis activity was detected at 28 °C in buffer (50 mM Tris–HCl, 300 mM NaCl, and 10% glycerol; pH = 8.0). (**E**) Lytic spectrum of LysO78. In (**C**,**E**), the decrease (%) in optical density at 600 nm (OD600) = (1 − absorbance of the bacterial suspension at the end of each treatment/absorbance at the beginning of each treatment) × 100%. Each data point and associated error bars correspond to the average of triplicate samples.

Sodium dodecyl sulfate polyacrylamide gel electrophoresis (SDS-PAGE) analysis indicated that the molecular weight of the purified LysO78 was around 34.0 kDa, and the value matched well with its predicted molecular weight (34.7 kDa) (Figure 6A). The lytic activities of LysO78 on viable cells of *E. coli* APEC O78 with different LysO78 concentrations were calculated in units/mg using a standard method published by Briers et al. [48]. The activity of LysO78 increased linearly up to 0.7 ng for LysO78, after which the activity gradually saturated (Figure 6B). The slope of the linear regression of this partial data set of the saturation curve was 0.2154 ΔOD600 nm/(min ng). According to the unit definition described, the specific activity of LysO78 was calculated as 64,620,000 units/mg (R^2^ = 0.9926) on *E. coli* APEC O78, corresponding to a 166-times higher activity compared with the broad-spectrum endolysin EL188 [59].

Lytic activity against the *E. coli* APEC O78 was observed in the pH range of 3.0–12.0, with an optimal pH of 8.0 (Figure 6C). LysO78 also exhibited a high lytic activity against the pathogenic *E. coli* APEC O78 in the pH range of 5.0–10.0. The lytic activity gradually decreased along with the treatment temperature increasing from 37 to 85 °C (Figure 6D). The endolysin LysO78 maintained a relatively high lytic activity after treatment at 4–55 °C for 1 h, while the lytic activity was drastically reduced at 65–85 °C for 1 h. The optimum reaction temperature of LysO78 was 28 °C, and the endolysin maintained 34.4% of its maximum reaction activity when tested at 55 °C (Figure S4). LysO78 also exhibited a high lytic activity against the pathogenic *E. coli* APEC O78 at 4 and 45 °C, with about 81.0% and 88.4% of its maximum reaction activity at 28 °C, respectively.

Lytic spectrum analysis showed that the endolysin LysO78 had broad-spectrum lytic activity against seven *E. coli* strains, five *Klebsiella* strains, five *Salmonella* strains, two *Shigella* strains, three *Burkholderia* strains, two *Pseudomonas* strains, two *R. solanacearum* strains, three *Yersinia* strains, one *A. baumannii* strain, one *M. smegmatis* strain, *C. arctica* R3-44^T^, and one *Bacillus cereus* strain (Figure 6E and Table 1). Among the tested Gram-negative strains, the endolysin LysO78 exhibited its highest lytic activity against *B. susongensis* L226, followed by *K. mobilis*, *P. syringae* 2779, and *Shigella* sp. The endolysin LysO78 also showed good lytic activity against *E. coli* strains, including *E. coli* EHEC O157:H7, *Klebsiella* strains, and *Salmonella* strains. The endolysin LysO78 could also lyse the tested Gram-positive strain *B. cereus* 411A, albeit with relatively low lytic activity, as the data of the negative control without enzyme were subtracted. In addition, we tested the activity of endolysin LysO78 on 18 Gram-positive strains, but none showed signs of lysis, including *Bacillus cereus* (ATCC10987), *Bacillus anthracis*, *Bacillus subtilis* 168, *Listeria monocytogenes*, *Streptococcus mutans*, *Streptococcus bovis*, *Streptococcus equi* subsp. *zooepidemicus*, *Streptococcus sanguinis*, *Streptococcus salivarius*, *Streptococcus pyogenes*, *Streptococcus agalactiae*, *Staphylococcus epidermidis*, and six *Staphylococcus aureus* strains. Therefore, the endolysin LysO78 was active against a number of Gram-negative pathogenic bacteria.

## 4. Discussion

In this study, we found a novel phage, vB_EcoM_APEC, with the ability to infect the poultry pathogenic *E. coli* APEC O78 strain. The genome of phage vB_EcoM_APEC exhibited low similarity to those of known phages, and most ORFs were hypothetical proteins, while the annotated ORFs also exhibited low similarity to known proteins. The conserved proteins (major capsid protein and terminase large subunit) of phage vB_EcoM_APEC had low identity with those of known phages. In addition to *E. coli* strains, phage vB_EcoM_APEC could also surprisingly lyse pathogenic *K. pneumoniae*. Generally speaking, most phages are highly host-specific and typically only infect and kill an individual species or even subspecies of bacteria. However, there are exceptions, and it is clear that some bacteriophages do productively infect a range of bacterial species belonging to different genera, families, orders, and classes [60,61,62]. *E. coli* and *K. pneumoniae* both belong to the family *Enterobacteriaceae*. Therefore, it seems reasonable to assume that phage vB_EcoM_APEC could infect *E. coli* and *K. pneumoniae.* Broad-host-range bacteriophages may promote genetic diversity and genetic exchange in microbial communities. The potential to infect a variety of alternate bacterial host species would maximize opportunities for effective viral reproduction in complex natural communities and biofilms, since bacteriophages that are able to capitalize on a wider variety of potential hosts would be more likely to encounter suitable prey and replicate under the prevalent in situ conditions. The vB_EcoM_APEC was the first reported *Chitinomonas-*associated bacteriophage, and it had a broad-host-range. Thus, this finding concerning phage vB_EcoM_APEC enriches our understanding of broad-host-range phages.

We predicted 11 prophage-like regions (Table S3) of *C. arctica* R3-44^T^ using the online server PHASTER [63]. Phage vB_EcoM_APEC was isolated from the MMC-induced supernatant of *C. arctica* R3-44^T^ and formed phage plaques on *E. coli* APEC O78 double-layer plates. Similar phage morphology of vB_EcoM_APEC has been observed in the MMC-induced supernatant of *C. arctica* R3-44^T^. As the PCR results from Figure S3 showed, 16 colonies were detected in the PCR product bands, and the sequences of the PCR products matched those of major capsid genes of phage vB_EcoM_APEC. At present, no phage plaques of vB_EcoM_APEC were observed on *C. arctica* R3-44^T^ double-layer plates. We speculated that this phenomenon might be due to the following reasons. As shown in Figure S1, the lysate of the R3-44^T^ strain was a mixture consisting of several induced phage particles. Phage vB_EcoM_APEC might be a latent phage in the genome of the strain, and it is possible that the induction of mitomycin caused the formation of diverse latent prophages in the bacterial genome. Phage vB_EcoM_APEC might be released along with other phages. Furthermore, numerous factors are reported to influence the infection ability of the phage, including the medium conditions, the existence of a resistance system in the bacterial genome, and the ability of the phage or lysin to access functional components in bacterial cells [64,65,66,67,68,69]. As for the test in this study, the adopted conditions might not have been suitable for phage infection.

Endolysins are widely believed to be novel antimicrobial agents because of their ability to lyse bacterial cells. Phage vB_EcoM_APEC is likely a temperate phage and may not be a good candidate for phage therapy. Therefore, further research on the endolysin of phage vB_EcoM_APEC is necessary. We identified and purified the novel endolysin of phage vB_EcoM_APEC, named LysO78. LysO78 is an α-helical protein consisting of two domains and two glutamic acids (E54, E64), which serve as the catalytic residues. It was also surprising that LysO78 was encoded on the genome of phage vB_EcoM_APEC induced from *Chitinomonas,* and that it could lyse *E. coli*. Besides *E. coli* strains, endolysin LysO78 exhibited broad bacteriolytic activity against a number of Gram-negative and one Gram-positive bacteria. A similar phenomenon was also found for *A. baumannii* phage ϕAB2 endolysin LysAB2, D2 endolysin Abtn-4, and PD-6A3 endolysin Ply6A3 [51,70]. As the genome phylogenic tree indicated that phage vB_EcoM_APEC was closer to the *Acinetobacter baumannii* phage, we speculate that a broad bactericidal spectrum may be a characteristic of the *A. baumannii* phage endolysin. The endolysin LysO78 can lyse zoonotic pathogens (*Salmonella*, *Shigella*, *Klebsiella*, *Escherichia*, *Yersinia*, and *A. baumannii*) and economical crops pathogens (*Ralstonia solanacearum* and *P. syringae*). In addition to its broad-spectrum bactericidal activity, LysO78 exhibited high lytic activity against pathogenic bacteria over wide pH and temperature ranges. The above advantages suggest that the endolysin LysO78 might be a promising candidate therapeutic agent for addressing multidrug-resistant pathogen infections in both agricultural and clinical settings.

## Figures and Tables

**Table 1:** The host range of the phage vB_EcoM_APEC and the lytic spectrum of endolysin LysO78.

Preservation Number	Strains	EOP of Phage vB_EcoM_APEC	LysO78
ATCC 13076	*Salmonella enterica*	−	+
CCTCC AB200057	*Salmonella* Saintpaul	−	+
CCTCC AB200056	*Salmonella* Sandiego	−	+
BNCC 186354	*Salmonella choleraesuis*	−	+
CCTCC AB2014173	*Salmonella* Typhimurium TA97	−	+
CCTCC AB204063	*Salmonella* Typhimurium TA100	−	−
CCTCC AB2014174	*Salmonella* Typhimurium TA102	−	−
CCTCC AB2013094	*Salmonella* Paratyphi CMCC(B)50094	−	−
CCTCC AB200060	*Shigella sonnei*	−	−
CCTCC AB2013093	*Shigella* sp.	−	+
CCTCC AB200059	*Shigella boydii*	−	−
BNCC 103609	*Shigella dysenteriae*	−	+
CCTCC AB2012147	*Klebsiella pneumoniae* D2	−	+
CCTCC AB208106	*Klebsiella pneumoniae* L17	−	+
CCTCC AB 200063	*Klebsiella pneumoniae* *subsp. ozaenae*	51.9%	+
CCTCC AB206144	*Klebsiella alba* CW-D3T	−	+
CCTCC AB91102	*Klebsiella mobilis*	−	+
CCTCC2010358	*Klebsiella oxytoca* PYR-1	−	−
CCTCC S2015201	*Pseudomonas arsenicoxydans*	−	+
CGMCC1.33331	*Pseudomonas syringae* 2779	−	+
	*Escherichia coli* APEC O78	100.0%	+
	*Escherichia coli* CC11	−	+
	*Escherichia coli* K12	76.7%	+
	*Escherichia coli* O157	−	−
NKCCMRNK7.C7303	*Escherichia coli* C7303	−	+
NKCCMRNK7.C7309	*Escherichia coli* C7309	−	+
NKCCMRNK7.C7341	*Escherichia coli* C7341	−	+
BNCC 192101	*Escherichia coli* EHEC O157:H7	−	+
	*Acinetobacter baumannii* Aba02	−	+
	*Yersinia pseudotuberculosis* ip95323	−	+
	*Yersinia pseudotuberculosis* type III	−	+
	*Yersinia enterocolitica* ye3	−	+
CCTCCAB2017239	*Ralstonia solanacearum* JW-95	−	+
CGMCC 1.12711	*Ralstonia solanacearum* FJAT-91	−	+
CCTCCAB2014142	*Burkholderia susongensis* L226	−	+
ATCC 29196	*Burkholderia glathei* N 16	−	−
CCTCCAB2014336	*Burkholderia anthina* XTB-5	−	+
CCTCCAB2010354	*Burkholderia zhejiangensis* OP-1	−	−
CCTCCAB2016346	*Burkholderia glumae* 9512	−	−
CCTCCAB2016347	*Burkholderia plantarii* 9509	−	+
	*Mycobacterium smegmatis* mc2 155	−	+
CCTCC PA2018059	*Mycobacterium neoaurum*	−	−
CCTCC AB 2010422	*Chitinimonas arctica* R3-44^T^	−	+
KCTC 22839	*Chitinimonas viridies* HMD2169^T^	−	−
	*Bacillus cereus* 411A	−	+
ATCC 10987	*Bacillus cereus*	−	−
CMCC63605	*Bacillus anthracis*	−	−
	*Bacillus subtilis* 168	−	−
CCTCC AB209106	*Listeria monocytogenes*	−	−
CCTCC AB99010	*Streptococcus mutans*	−	−
CCTCC AB2016240	*Streptococcus bovis*	−	−
CCTCC AB204053	*Streptococcus equi* subsp. *zooepidemicus*	−	−
CCTCC AB99004	*Streptococcus sanguinis*	−	−
CCTCC PB2020011	*Streptococcus agalactiae*	−	−
CCTCC PB2020002	*Streptococcus pyogenes*	−	−
CCTCC AB99008	*Streptococcus salivarius*	−	−
CCTCC PB2018375	*Staphylococcus epidermidis*	−	−
BNCC 134242	*Staphylococcus aureus* subsp.* aureus*	−	−
CCTCC AB2010022	*Staphylococcus aureus*	−	−
CCTCC AB2010021	*Staphylococcus aureus*	−	−
CCTCC PB2018158	*Staphylococcus aureus*	−	−
CCTCC PB2020004	*Staphylococcus aureus*	−	−
CCTCC AB204036	*Staphylococcus aureus*	−	−

For efficiency of plating (EOP) of phage vB_EcoM_APEC, a percentage indicates the phage could infect the specified bacteria and generate plaques efficiently, and − indicates the phage did not generate plaques with respect to the specified bacteria. Under LysO78, the specified bacteria were (+) or were not (−) lysed by endolysin LysO78.

## Data Availability

The GenBank accession number of phage vB_EcoM_APEC is MT664721.

## Supplementary Materials

The following are available online at https://www.mdpi.com/article/10.3390/v13061034/s1. Table S1: All predicted open reading frames (ORFs) for bacteriophage vB_EcoM_APEC. Table S2: Oligonucleotide primers pairs used in this study of endolysin LysO78 and mutants. Table S3: The predicted prophages of *Chitinimonas arctica* R3-44^T^. Figure S1: The induced prophage morphology of *Chitinimonas arctica* R3-44^T^ under TEM. Figure S2: Phage morphology under TEM. Figure S3: PCR detection of the major capsid gene in *Chitinimonas arctica* R3-44^T^. Figure S4: The influence of reaction temperature on endolysin LysO78.
