# Supplementary material for: Genomic Analysis of a Novel Phage Infecting the Turkey Pathogen Escherichia coli APEC O78 and Its Endolysin Activity
Source: Viruses. 2021 May 31;13(6):1034. doi: 10.3390/v13061034 (PMC8229158; doi:10.3390/v13061034)
Supplement: Supplementary file 1 [file viruses-13-01034-s001.zip › viruses-1140211-supplementary.pdf]

Table S1. All predicted open reading frames (ORFs) for bacteriophage vB_EcoM_APEC.

ORF	Start	End	Length (bp)	Protein mass (kDa)	Product
1	35	448	414	15.51	Hypothetical protein
2	451	906	456	15.72	Hypothetical protein
3	910	2949	2040	74.55	Hypothetical protein
4	2946	5558	2613	93.13	Lytic transglycosylase
5	5780	8089	2310	84.23	Minor capsid protein
6	8281	8673	393	15.11	Hypothetical protein
7	8673	10,346	1674	63.27	DNA-packaging protein
8	10,358	12,787	2430	86.74	Tail fiber protein
9	12,868	13,206	339	12.58	Holin
10	13,190	13,474	285	10.61	Hypothetical protein
11	13,476	14,084	609	22.96	Endolysin
12	14,096	14,293	198	7.58	Hypothetical protein
13	14,290	14,490	201	7.92	Head portal-like protein
14	14,456	14,836	381	14.76	Hypothetical protein
15	14,836	15,189	354	13.07	Hypothetical protein
16	15,186	15,491	306	11.58	Small distal tail fiber subunit
17	15,534	15,710	177	6.85	Hypothetical protein
18	15,719	16,330	612	22.91	Phage-type exonuclease
19	16,330	17,028	699	25.88	Hypothetical protein
20	17,102	17,260	159	6.19	Hypothetical protein
21	17,250	17,609	360	13.44	Hypothetical protein
22	17,606	17,968	363	14.53	Hypothetical protein
23	17,965	18,351	387	14.64	Hypothetical protein
24	18,339	18,866	528	19.93	Hypothetical protein
25	19,057	19,497	441	16.85	HNH endonuclease
26	19,494	19,754	261	9.60	Hypothetical protein
27	20,073	20,258	186	7.15	Hypothetical protein
28	20,255	20,890	636	24.67	Hypothetical protein
29	20,887	21,078	192	7.41	Hypothetical protein
30	21,207	21,437	231	8.84	Prophage anti-repressor
31	21,430	21,642	213	8.47	Tail fiber protein
32	21,652	22,389	738	27.99	Replication protein O
33	22,389	23,693	1305	48.56	Replicative DNA helicase
34	23,878	24,066	189	7.33	Hypothetical protein
35	24,110	24,280	171	6.89	Hypothetical protein
36	24,447	24,713	267	9.98	Hypothetical protein
37	24,706	24,918	213	8.05	Hypothetical protein
38	24,983	25,162	180	6.90	Ribonuclease H
39	25,149	25,550	402	15.45	Hypothetical protein
40	25,761	26,726	966	37.51	Repressor
41	26,786	27,058	273	10.63	Hypothetical protein
42	27,148	27,780	633	24.65	HNH homing endonuclease
43	27,773	28,354	582	22.35	Terminase large subunit
44	28,341	28,718	378	13.94	Hypothetical protein
45	29,153	29,383	231	8.84	Hypothetical protein
46	29383	29574	192	6.59	Hypothetical protein
47	29,571	31,187	1617	60.60	Head to tail connecting protein
48	31,177	31,530	354	13.89	Sigma factor regulator

49	31,527	32,180	654	23.43	Capsid assembly protein
50	32,195	33,085	891	32.84	Major capsid protein
51	33,134	33,721	588	22.12	Tail tubular protein A
52	33,651	35,828	2178	80.17	Tail tubular protein B

Table S2. Oligonucleotide primers pairs used in this study of endolysin LysO78 and mutants.

Primer pairs	Sequence (5'–3')
E54A-For	TTGGCATgcaACTGCAAAAACCATGCAGCCAA
E54A-Rev	TTGCAGTtgcATGCCAAGTTGTAGCGAGCATG
E54Q-For	TTGGCATcagACTGCAAAAACCATGCAGCCAA
E54Q-Rev	TTGCAGTctgATGCCAAGTTGTAGCGAGCATG
E54D-For	CTTGGCATgatACTGCAAAAACCATGCAGCCA
E54D-Rev	TGCAGTatcATGCCAAGTTGTAGCGAGCATGT
E63A-For	GCAGCCAATTgcaGAATATGGAAAGGGTAAAGGCCG
E63A-Rev	ATTCtgcAATTGGCTGCATGGTTTTTGCAGTT
E63Q-For	GCAGCCAATTcagGAATATGGAAAGGGTAAAGGCCG
E63Q-Rev	ATTCctgAATTGGCTGCATGGTTTTTGCAGTT
E64A-For	GAAgcaTATGGAAAGGGTAAAGGCCGTCCTTA
E64A-Rev	CCCTTTCCATAtgcTTCAATTGGCTGCATGGTTTT
E64Q-For	GAAcagTATGGAAAGGGTAAAGGCCGTCCTTA
E64Q-Rev	CCCTTTCCATActgTTCAATTGGCTGCATGGTTTT
E54A/E63A-For	gcaaaaaccatgcagccaattgcaGAATATGGAAAGGGTAAAGGCCG
E54A/E63A-Rev	tggctgcatggtttttgcagttgcATGCCAAGTTGTAGCGAGCAT
E54Q/E63Q-For	gcaaaaaccatgcagccaattcagGAATATGGAAAGGGTAAAGGCCG
E54Q/E63Q-Rev	tggctgcatggtttttgcagtctgATGCCAAGTTGTAGCGAGCAT
E54A/E64A-For	caaaaaccatgcagccaattgaagcaTATGGAAAGGGTAAAGGCCGT
E54A/E64A-Rev	tggctgcatggtttttgcagttgcATGCCAAGTTGTAGCGAGCAT
E54Q/E64Q-For	caaaaaccatgcagccaattgaacagTATGGAAAGGGTAAAGGCCGT
E54Q/E64Q-Rev	tggctgcatggtttttgcagtctgATGCCAAGTTGTAGCGAGCAT

The lowercase characters marked in red indicate nucleotides corresponding to the mutated amino acid site(s).

Table S3. The predicted prophages of *Chitinimonas arctica* R3-44^{T}.

Region	Length (kb)	Completeness	Most Common Phage	Host strain	GenBank number
1	53.8	questionable	vB_EcoM_ECOO78	*E. coli* APEC O78	NC_041926
2	4.6	incomplete	Stx2a_F451	*E. coli* O157:H7	NC_049924
3	37.8	incomplete	vB_EcoM_ECOO78	*E. coli* APEC O78	NC_041926
4	30.8	intact	MD8	*Pseudomonas aeruginosa*	NC_031091
5	34	intact	RSA1	*Ralstonia solanacearum*	NC_009382
6	17.5	incomplete	phiPSA1	*Pseudomonas syringae*	NC_024365
7	26	intact	vB_EcoM_ep3	*E. coli* CC11	NC_025430
8	36.4	incomplete	phiPSA1	*Pseudomonas syringae*	NC_024365
9	13.9	incomplete	vB_EcoM_ECOO78	*E.coli* APEC O78	NC_041926
10	8.9	incomplete	SfIV	*Shigella flexneri*	NC_022749
11	29.1	intact	RSY1	*Ralstonia solanacearum*	NC_025115

Region: the number assigned to the region; Length: the length of the sequence of that region (in kb); Completeness: a prediction of whether the region contains an intact or incomplete prophage based

on the above criteria; Total Proteins: the number of ORFs present in the region; Most Common Phage: the phage(s) with the highest number of proteins similar to those in the region.

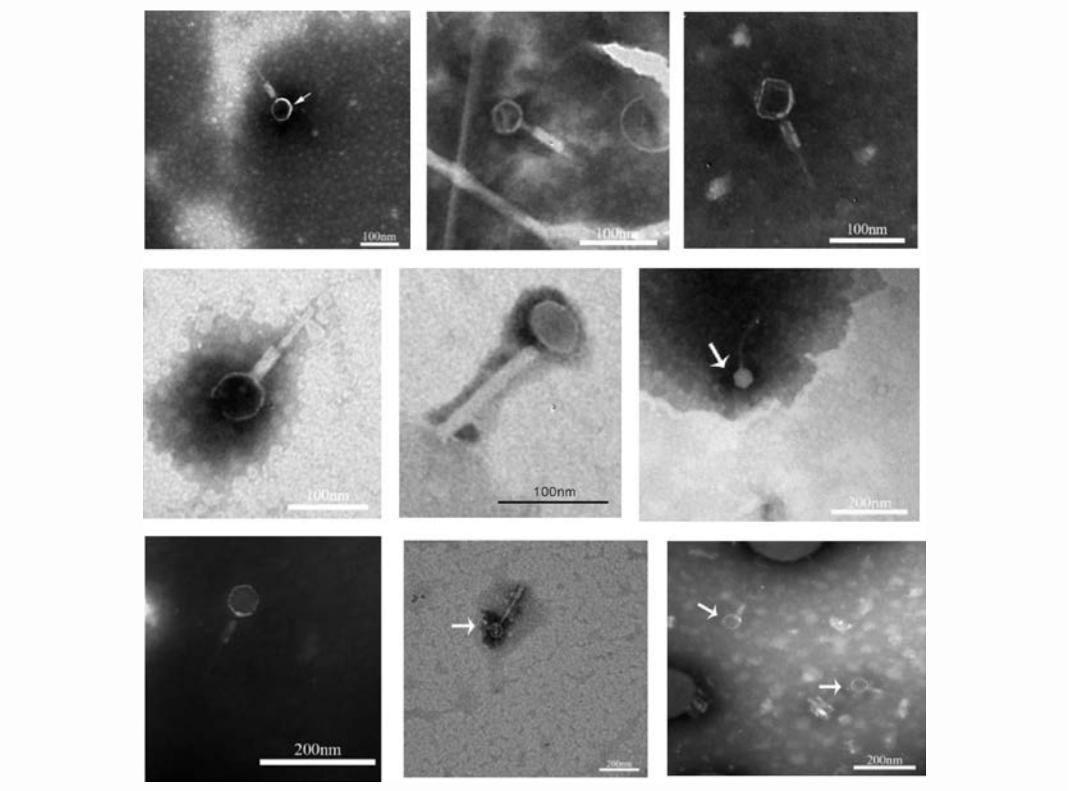

Figure S1. The induced prophage morphology of *Chitinimonas arctica* R3-44^{T} under TEM.

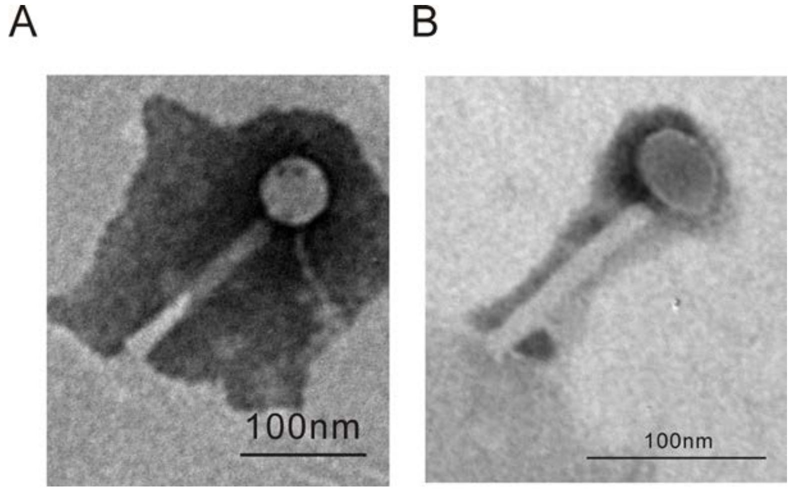

Figure S2. Phage morphology under TEM. (A) The phage morphology of vB_EcoM_APEC. (B) The phage morphology in the MMC–induced supernatant of *Chitinimonas arctica* R3-44^{T}.

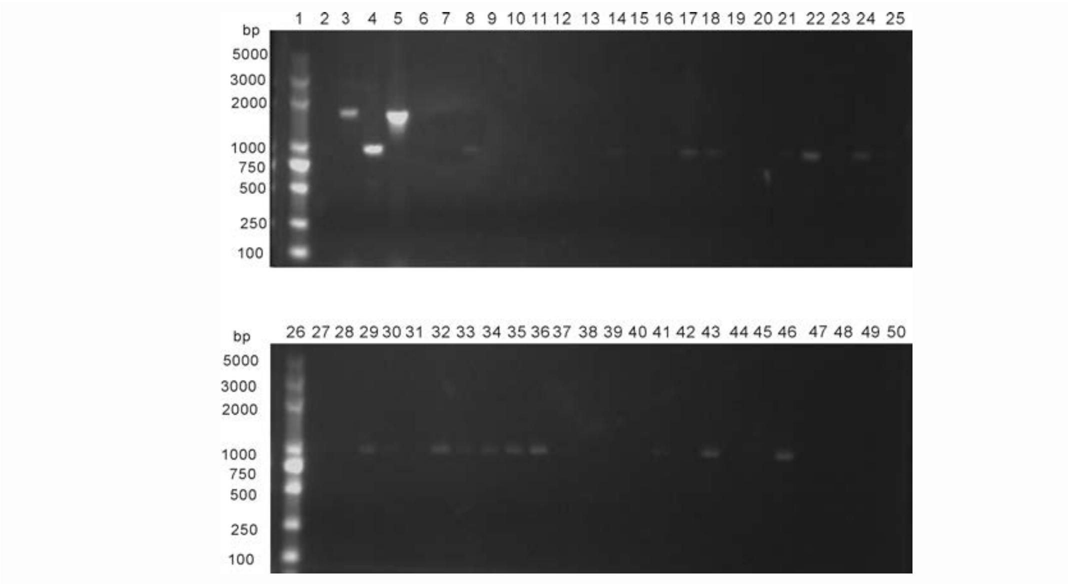

Figure S3. PCR detection of the major capsid gene in *Chitinimonas arctica* R3-44^{T}. Lane 1 and 26, DL5000 Marker; Lane 2, negative control; Lane 3, 16S rDNA from phage genome (residual genomic DNA of host strain *E. coli* APEC O78); Lane 4, positive control of major capsid gene; Lane 5, 16S rDNA from single colony of *Chitinimonas arctica* R3-44^{T}; Lanes 6–25 and 27–50, the detection of the major capsid gene from different colonies of *Chitinimonas arctica* R3-44^{T}. In order to evaluate DNA availability from different colonies, 16S rDNA was detected.

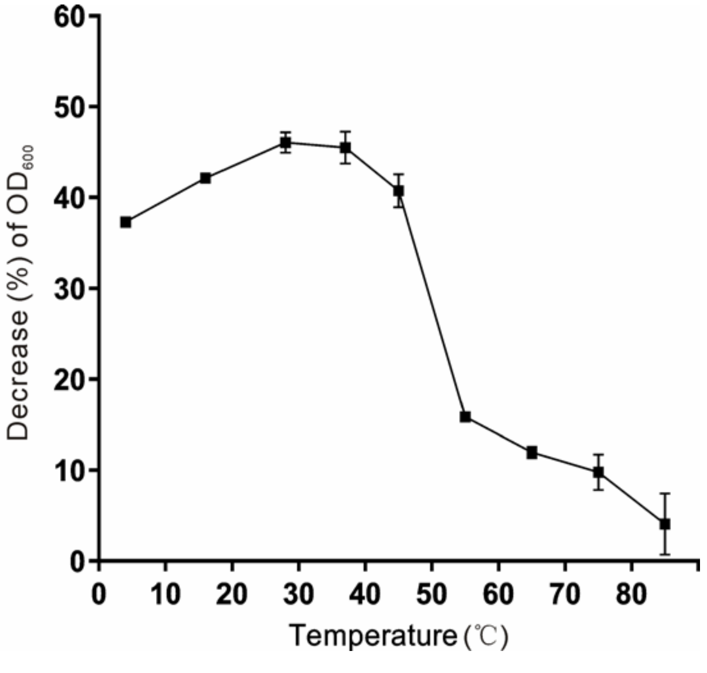

Figure S4. Influence of the reaction temperature on endolysin LysO78. The decrease (%) in optical density at 600 nm (OD_{600}) = (1− absorbance of the bacterial suspension at the end of each treatment/absorbance at the beginning of each treatment) × 100%.
